# Automatic Detection and Recognition of Pig Wasting Diseases Using Sound Data in Audio Surveillance Systems

**DOI:** 10.3390/s131012929

**Published:** 2013-09-25

**Authors:** Yongwha Chung, Seunggeun Oh, Jonguk Lee, Daihee Park, Hong-Hee Chang, Suk Kim

**Affiliations:** 1 Department of Computer and Information Science, College of Science and Technology, Korea University, Sejong 339-700, Korea; E-Mails: ychungy@korea.ac.kr (Y.C.); gmo85@korea.ac.kr (S.O.); eastwest9@korea.ac.kr (J.L.); 2 Department of Animal Science, Institute of Agriculture & Life Sciences, College of Agriculture and Life Sciences, Gyeongsang National University, Jinju 660-701, Korea; E-Mail: hhchang@gnu.ac.kr; 3 College of Veterinary Medicine, Gyeongsang National University, Jinju 660-701, Korea; E-Mail: kimsuk@gnu.ac.kr

**Keywords:** pig wasting diseases, sound data, mel frequency cepstrum coefficient, support vector data description, sparse representation classifier

## Abstract

Automatic detection of pig wasting diseases is an important issue in the management of group-housed pigs. Further, respiratory diseases are one of the main causes of mortality among pigs and loss of productivity in intensive pig farming. In this study, we propose an efficient data mining solution for the detection and recognition of pig wasting diseases using sound data in audio surveillance systems. In this method, we extract the Mel Frequency Cepstrum Coefficients (MFCC) from sound data with an automatic pig sound acquisition process, and use a hierarchical two-level structure: the Support Vector Data Description (SVDD) and the Sparse Representation Classifier (SRC) as an early anomaly detector and a respiratory disease classifier, respectively. Our experimental results show that this new method can be used to detect pig wasting diseases both economically (even a cheap microphone can be used) and accurately (94% detection and 91% classification accuracy), either as a standalone solution or to complement known methods to obtain a more accurate solution.

## 1. Introduction

Early detection of health anomalies is an important issue in the management of group-housed livestock. In particular, the vocalization of sick pigs could indicate respiratory diseases, which are one of the main causes of mortality among pigs and loss of productivity in intensive pig farming [1]. To minimize the damage caused by various respiratory diseases, it is necessary to develop technology for collecting and analyzing livestock data.

Coughing is one of the symptoms and a central element in the screening and diagnosis of common respiratory diseases. It is one of the body's defense mechanisms against respiratory infections, a sign of disorder. Coughing is presented by a sudden expulsion of air from the airways, which is characterized by a typical sound. This sound is so characteristic that it allows identification of the cough, distinguishing it from other vocal manifestations [1]. Clinically, coughing is the most frequent presenting symptom of many diseases affecting airways and lungs, and is often an early symptom of some respiratory diseases [2].

Sound analysis is of considerable importance, because it helps to classify and quantify coughs. Sound production by animals is a candidate bio-signal that can be measured easily at a distance, thereby not causing any additional stress to the animals [3]. Furthermore, in recent years, sound analysis has become an increasingly important tool to interpret the behavior, health condition, and well-being of animals.

Along with monitoring animals using wireless sensor network technology [4–6], a rich variety of studies have been reported on sound analysis: applied to animal sounds in general [7–9], and to farm animals [10–13] and pigs in particular [3,7,12,14–18]. The bio-acoustic study by Gutierrez *et al.* [1] was aimed at classifying the different pig wasting diseases through sound analysis with emphasis given to differences in the acoustic footprints of coughs, and they stated that their study could be useful in supporting an early detection method based on the on-line cough counter algorithm for the initial diagnosis of sick animals in breeding farms. In fact, several excellent studies were proposed for the detection of cough sounds in a pig house [7,15,19,20]. For example, Hirtum and Berckmans performed some experiments under laboratory conditions and introduced algorithms to detect cough sounds and to classify the animals as ill or healthy [7]. Guarino *et al.* detected different cough events in field conditions with an accuracy of 86.2% using a simple algorithm, and by applying on-line monitoring of continuous sound registration [20]. Exadaktylos *et al.* extended existing cough identification methods and proposed a real-time method for identifying sick pig cough sounds [19]. However, we still require further elaboration to this detection problem and recognition of pig wasting diseases using sound data in audio surveillance systems.

In this study, we introduce an efficient data mining solution for the detection and recognition of pig wasting diseases using sound data in audio surveillance systems. Primarily, pig sound acquisition process is performed, and the most widely used feature of sound analysis, the Mel Frequency Cepstrum Coefficient (MFCC), is extracted in the data preprocessing phase. The Support Vector Data Description (SVDD), which is often called an anomaly or novelty detector, naturally detects pig wasting diseases by using sound data. Finally, the Sparse Representation Classifier (SRC) classifies pig wasting diseases into Postweaning Multisystemic Wasting Syndrome (PMWS), Porcine Reproductive and Respiratory Syndrome (PRRS) virus and Mycoplasma Hyopneumoniae (MH), which are the major pathogenic diseases in farms. To the best of our knowledge, this is the first report of the detection and recognition of pig wasting diseases by using sound data, and SVDD/SRC learning for early anomaly detection and identification, respectively. Moreover, the application of a data mining method is appropriate considering the continuous and large incoming data stream that is a characteristic of audio surveillance systems for a pig house. The performance of the proposed system is validated via experiments, which confirm that its various measures are satisfactory (94% detection and 91% classification accuracy). The rest of this paper is organized as follows: Section 2 describes the proposed pig wasting disease detection and recognition system with some background concepts. The simulation results are presented in Section 3, followed by the conclusions in Section 4.

## 2. Pig Wasting Disease Detection and Recognition System

In this section, we summarize the pig sound acquisition process, the MFCC, the basic concept of the SVDD to detect pig wasting diseases, and the SRC to identify the types of pig wasting diseases in detail: PMWS, PRRS, and MH. We also introduce the proposed pig wasting disease detection and recognition system based on the hierarchical structure of SVDD and SRC. At the first level, an SVDD distinguishes cough sounds due to pig wasting diseases from normal sounds. At the second level, a SRC classifies the incoming audio signal into three types of pig wasting diseases: PMWS, PRRS, and MH.

### 2.1. Pig Sound Acquisition Process and Mel Frequency Cepstrum Coefficients (MFCC)

The pig sound acquisition process can be used to distinguish pig sounds (coughs, screams, sneezes, grunts, *etc.*) from noise (foot sounds, ventilation sounds, *etc.*) in a pig house. It can improve the recognition ratio under various types of background noises, and reduce the computing power waste induced by incorrect sound detection. In this study, we use a pig sound acquisition process that is based on the Jan čovi č's detection algorithm [21] reinforced with a short-time energy against noise sound.

In this study, the MFCC algorithm is used for the feature extraction module. The Mel frequency scale is the most widely used feature in sound analysis, with its simple calculation, good distinction ability, anti-noise capability, and other advantages [22].

**Figure 1.**
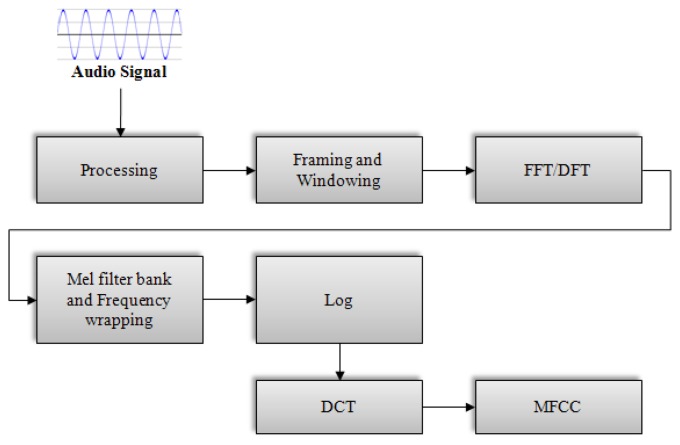
Flowchart of MFCC extraction procedure.

A block diagram of the structure of an MFCC processor is shown in Figure 1. In the first step, the continuous audio signal is blocked into frames of *N* samples, with adjacent frames being separated by *M* (*M* < *N*). Typical values for *N* and *M* are *N* = 256 and *M* = 100. The next step in the processing is to window each individual frame to minimize the signal discontinuities at the beginning and end of each frame. Typically, the Hamming window is used. In the next processing step, the Fast Fourier Transform is used to convert each frame of *N* samples from the time domain into the frequency domain. The scale of frequency is then converted from the linear to mel scale. Then, the logarithm is taken from the results. In the final step, the log mel spectrum is converted back to the time domain, resulting in the MFCC.

### 2.2. Support Vector Data Description (SVDD)

In recent times, the support vector learning method has reached maturity as a viable tool in the area of intelligent systems [23]. Among the important application areas for support vector learning are the one-class classification problems. In one-class classification problems, only the training data for the normal class are generally given, and after the training phase is finished, we are required to decide whether each test vector belongs to the normal or abnormal class. One-class classification problems are often called outlier detection problems or novelty detection problems. One of the most well-known support vector learning methods for the one-class problems is the SVDD.

The SVDD method, which approximates the existence area of objects belonging to a normal class, is derived as follows [23]: Consider a ball *B* with center *a* ∈ *R^d^* and radius *R*, and the training data set *D* consisting of objects *x_i_* ∈ *R^d^*, *i* = 1, …,*N*. It should be noted that, since the training data are usually prone to noise, some part of the training data *D* could consist of abnormal objects. The main idea of the SVDD is to find a ball that can achieve the two conflicting goals simultaneously: the ball should be as small as possible, and, more importantly, it should contain as many training data as possible. Obviously, some balls that meet these multiple objectives may be obtained by solving the optimization problem:

(1) $$\begin{array}{c} \min L_{o}(R^{2},a,\xi )=R^{2}+C\overset{N}{\underset{i=1}{\text{∑}}}\xi_{i}\\ \text{s}.\text{t}.∥x_{i}-a{∥}^{2}\leq R^{2}+\xi_{i},\xi_{i}\geq 0,\forall i. \end{array}$$

Here, the slack variable *ξ_i_* represents the penalty associated with the deviation of the *i* – *th* training pattern outside the ball. The objective function of the above optimization problem consists of two conflicting terms: the square of radius *R*^2^ and the total penalty $\sum_{i=1}^{N}\xi_{i}$. The constant *C* controls the relative importance of each term, and is thus called the trade-off constant. The above dual problem can be derived as follows. First, by introducing a Lagrange multiplier for each inequality condition, we obtain the Lagrange function:

$$\begin{array}{l} \text{L}=R^{2}+C\overset{N}{\underset{I=1}{\text{∑}}}\xi_{i}\\ +\overset{N}{\underset{i=1}{\text{∑}}}\alpha_{i}\;[{\left(x_{i}-a\right)}^{T}(x_{i}-a)-R^{2}-\xi_{i}]-\overset{N}{\underset{i=1}{\text{∑}}}\eta_{i}\xi_{i} \end{array}$$

where *α_i_* ≥ 0, *η_i_* ≥ 0, ∀*i*.

From the saddle point condition, the optimal solution of Equation (1) should satisfy:

(2) $$\begin{array}{l} \frac{\partial L}{\partial (R^{2})}=0:\overset{N}{\underset{i=1}{\text{∑}}}\alpha_{i}=1.\\ \frac{\partial L}{\partial a}=0:a=(\underset{i}{\text{∑}}\alpha_{i}x_{i})/\underset{i}{\text{∑}}\alpha_{i}=\overset{N}{\underset{i=1}{\text{∑}}}\alpha_{i}x_{i}\\ \frac{\partial L}{\partial \xi_{i}}=0:\alpha_{i}\in [0,C],\forall \text{i}. \end{array}$$

With the substitution of the above into *L*, the Lagrange function can be expressed in terms of the dual variables as follows:

$$\begin{array}{c} L=\sum_{i=1}^{N}\alpha_{i}<x_{i},x_{i}>-\sum_{i=1}^{N}\sum_{j=1}^{N}\alpha_{i}\alpha_{j}<x_{i},x_{j}>,\\ \text{where}\sum_{i=1}^{N}\alpha_{i}=1,\alpha_{i}\in [0,C],\forall i. \end{array}$$

Thus, the dual problem can be written as:

(3) $$\begin{array}{l} \max_{\alpha }\overset{N}{\underset{i=1}{\text{∑}}}\alpha_{i}<x_{i},x_{j}>-\overset{N}{\underset{i=1}{\text{∑}}}\overset{N}{\underset{j=1}{\text{∑}}}\alpha_{i}\alpha_{j}<x_{i},x_{j}>\\ \text{s}.\text{t}.\overset{N}{\underset{i=1}{\text{∑}}}\alpha_{i}=1,\alpha_{i}\in [0,C],\forall i. \end{array}$$

It should be noted that Equation (3) is equivalent to the quadratic programming (QP) problem:

(4) $$\begin{array}{l} \min_{\alpha }\overset{\text{N}}{\underset{i=1}{\text{∑}}}\overset{\text{N}}{\underset{j=1}{\text{∑}}}\alpha_{i}\alpha_{j}<x_{i},x_{j}>-\overset{\text{N}}{\underset{i=1}{\text{∑}}}\alpha_{i}<x_{i},x_{i}>\\ \text{s}.\text{t}\overset{N}{\underset{i=1}{\text{∑}}}\alpha_{i}=1,\alpha_{i}\in [0,C],\forall i. \end{array}$$

In addition, it should be noted that from the Kuhn-Tucker complementarity condition, it should hold true that:

(5) $$\alpha_{i}\;(\left\|x_{i}-a\right\|-R^{2}-\xi_{i})=0,\forall i.$$

From the above, we can easily show that ultimately only the data points on the boundary or outside the ball can have positive alpha values. These data points are called the support vectors. Once the *α_i_* are obtained by solving the problem (4), the optimal center is given by Equation (2). In addition, the optimal value of *R*^2^ is acquired by applying condition (5) to the support vectors. After the training phase has been completed, we decide whether a given test point *x* ∈ ℝ^d^ belongs to the normal class utilizing the criterion:

$$\begin{array}{cc} f(x) & =R^{2}-{\left\|x-a\right\|}^{2}\\ & =R^{2}-<x,x>-2\overset{\text{N}}{\underset{i=1}{\text{∑}}}\alpha_{i}<x_{i},x>+\overset{\text{N}}{\underset{i=1}{\text{∑}}}\overset{\text{N}}{\underset{j=1}{\text{∑}}}\alpha_{i}\alpha_{j}<x_{i},x_{j}>\\ & \geq 0 \end{array}$$

### 2.3. Sparse Representation Classifier (SRC)

Sparse Representation (SR) was first proposed for signal representation. In the past few years, SR has successfully applied in many practical applications such as signal compression and coding, image de-noising, and compressive sensing. In recent years, several supervised classification methods based on sparse representation, viz. sparse representation classifier (SRC), have been proposed [24–27].

The classification problem based on sparse representation can be formulated as follows [24–27]: Suppose that we have *n* training samples for *c* classes and sufficient training samples belong to the *k* – *th* class, *A_K_* = [*x_k_*_,1_, *x_k_*_,2_,⋯, *x_k,n_k__*] ∈ ℝ*^m×n_k_^*, where *m* is the dimension of samples and *n_k_* is the number of training samples of the *k* – *th* class. Any test sample *y* ∈ ℝ*^m^* from the *k* – *th* class can be approximately represented as the linear combination of training samples of this class:

(6) $$y=\alpha_{k,1}x_{k,1}+\alpha_{k,2}x_{k,2}+\cdots \alpha_{k,n_{k}}x_{k,n_{k}}$$

Since the label of *y* is unknown initially, we present *y* as the linear combination of all the training samples:

(7) $$y=A\alpha_{0}$$

where *A* = [*A*_1_, *A*_2_,⋯, *A*_c_] = [*x*_1,1_, *x*_1,2_,⋯, *x_c,n_c__*] ∈ ℝ*^m×n^* is a matrix composed of all the *n* training samples of *c* classes and *α*_0_ = [0,⋯,0, *α_k_*_,1_, *α_k_*_,2_,⋯, *α_k,n_k__*,0,⋯,0]*^T^* ∈ ℝ*^m×n^* is the coefficient vector whose nonzero entries are only associated with the *k*–*th* class. When *c* is large, *α*_0_ will be sparse.

If m < *n*, Equation (7) is underdetermined. The problem of sparse representation is to search a vector α such that Equation (7) is satisfied and ‖*α*‖_0_ is minimized, where ‖*α*‖_0_ is the *l*_0_–*norm* of α. This can be described as

(8) $${\hat{\alpha }}_{0}=\text{arg}\underset{\alpha }{\min}{\left\|\alpha \right\|}_{0}\;\text{subject to}\;y=A\alpha$$

However, finding the sparse solution of Equation (8) is NP-hard: namely there is no known procedure for obtaining the sparsest solution, which is significantly more efficient than exhausting all subsets of the entries for *α*. The theory of sparse representation and compressive sensing reveals that we can solve the following convex relaxed optimization to obtain approximate solution:

(9) $${\hat{a}}_{1}=\text{arg}\underset{\alpha }{\min}{\left\|\alpha \right\|}_{1}\;\text{subject to}\;y=A\alpha$$

where ‖*α*‖_1_ is the *l*_1_–*norm* of *α*.

This problem can be solved in polynomial time by standard linear programming methods. Given a new test data *y* from one of the classes in the training set, its sparse representation *α̂*_1_ is computed by Equation (9). The nonzero entries in the estimate *α̂*_1_will be associated with the columns of *A* from a single object class *i*, and we can easily assign the test sample *y* to that class.

In the context of pig wasting disease sound classification, the training data and test data might be taken under different conditions, and it may lead to nonzero entries associated with multiple object classes. Therefore, in general, the system outputs the results in sorted order according to the score value. In this paper, we use the coefficient value in the solution *α̂*_1_ of *l*_1_ minimization problem as the score value. Algorithm 1 summarizes the pig wasting disease sound classification procedure.


**Algorithm 1.** Pig wasting disease sound classification procedure.
Input: a matrix of training samples*A* = [*A*_1_, *A*_2_,…, *A*_c_] ∈ ℝ*^m×n^* for *c* classes, a test sample *y* ∈ ℝ*^m^*.Normalize the columns of *A* to have unit *l*_2_ norm.Solve the *l*_1_ minimization problem: ${\hat{\alpha }}_{1}=\text{arg}\underset{\alpha }{\min}{\left\|\alpha \right\|}_{1}\;\text{subject to}\;y=A\alpha .$Compute the mean coefficient value of each class.
$mc_{\text{i}}=\frac{1}{n_{i}}\overset{n_{i}}{\underset{1}{\text{∑}}}\delta_{i}\;({\hat{\alpha }}_{1})\;\text{for}\;i=1,2,\ldots ,c$*mc*_i_: mean coefficient value of *i* – *th* class;*n*_i_: number of elements in *i* – *th* class;*δ_i_*(*α̂*_1_): characteristic function that selects the coefficients associated with the *i* – *th* class;Sort the class in descending order according with mean coefficient value of classes.Output: pig wasting diseases, which have large mean coefficient value.

### 2.4. Pig Wasting Disease Detection and Recognition System

**Figure 2.**
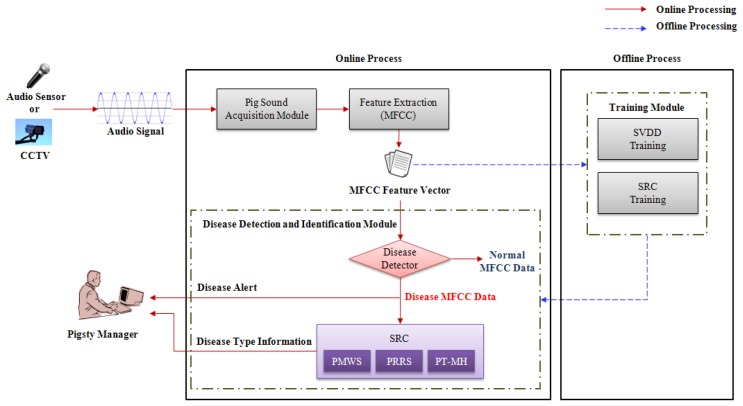
Overall structure of the pig wasting diseases detection and recognition system.

The proposed automatic pig wasting disease detection and recognition system is composed of four modules: the pig sound acquisition process, feature extraction and pig wasting disease detection and identification module that comprises three online process modules, and SVDD/SRC training module of an offline process module (see Figure 2). In the pig sound acquisition process, real pig sounds (coughs, screams, sneezes, grunts, *etc.*) are obtained from the audio sensor (PILLAR CM-5010Pro, Seoul, Korea) installed at the center-positioned 2 m height ceiling of a compartment in a pig house, which filters out the background noises (foot sounds, ventilation sounds, *etc.*). In the feature extraction module, the MFCC algorithm is used for the feature extraction from the audio signal via sound sensors or a CCTV camera. The SVDD/SRC training module performs training off-line based on the MFCC. In the pig wasting disease detection and identification module, the proposed mechanism is constructed as a hierarchical two-level structure.

## 3. Results

### 3.1. Data Collection and Data Sets

The experiment was conducted in a commercial swine production farm located in Chungnam Province, South Korea. In order to collect cough sounds emitted by infected pigs, a total of 36 pigs (Yorkshire × Landrace × Duroc) were used in this experiment with the average weight ranging between 25 and 30 kg. Twenty-two pigs were housed in a 1.8 m × 4.8 m size pen with a room temperature of about 23 °C. The blood samples of the suspected infected pigs were collected and subjected to serological analysis to determine PMWS, PRRS, and MH infections. The cough sounds emitted by infected pigs were recorded individually for 30 min depending on cough attacks using a digital camcorder (JVC GR-DVL520A, Yokohama, Japan), which was placed within a meter distance from the sick pigs. The observations were recorded under field conditions. Although pigs were allowed to move around the pen, most of our recordings were done when they were lying on the floor. The cough sounds emitted by each infected pig were recorded individually. The recorded signals were digitalized in a PC with a standard soundcard Realtek AC97 at 16 bits and 44.1 Hz sampling rates using Cool Edit (Adobe, San Jose, CA, USA) program. Using the pig sound acquisition process described in Section 2.1, the sounds collected were classified through a labeling method into PMWS, PRRS and MH infections. They were then used as reference data for the detection of pig wasting diseases (for more details refer to Gutierrez *et al.* [1]).

**Figure 3.**
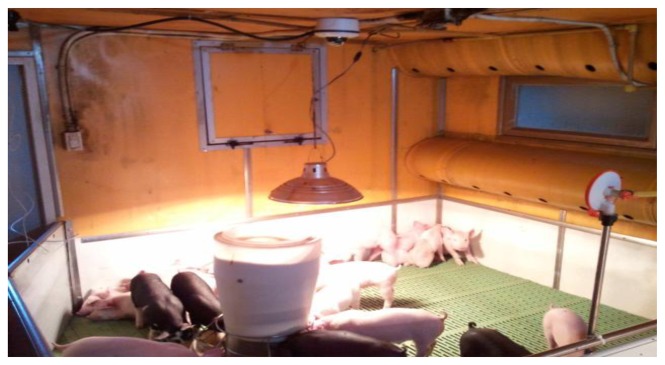
Picture of a pig house installed a stationary CCTV with an audio sensor.

**Figure 4.**
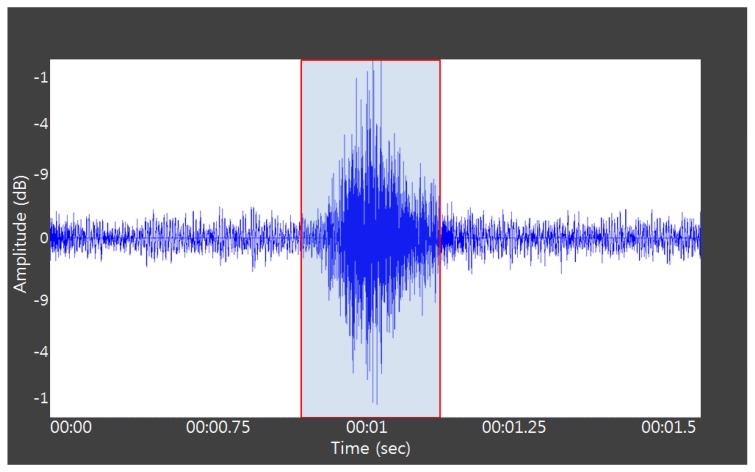
Typical example of automatic pig sound acquisition process marked using the red rectangle.

**Figure 5.**
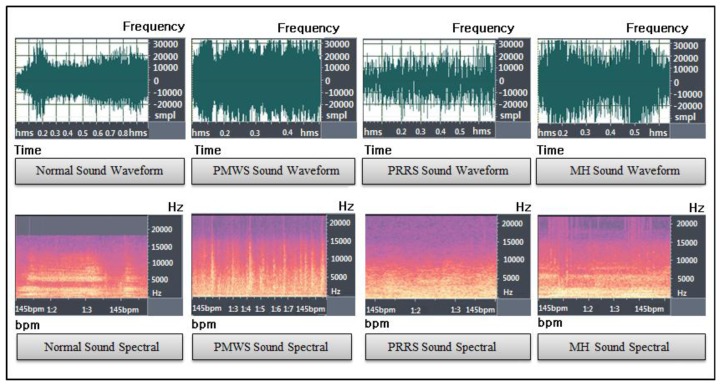
Waveforms and spectrograms of pig wasting diseases cough sound and normal sound samples.

Apart from the cough sounds due to pig wasting diseases, we collected pig sounds for a month between May and June in 2012 in a real pig house located in Jinju, Korea, which had a stationary CCTV with an audio sensor (PILLAR CM-5010Pro) installed at the center-positioned 2 m height ceiling of a compartment in it (see Figure 3). Then normal pig sounds (such as non-infectious coughs, screams, and grunts), excluding noisy data, were acquired using the pig sound acquisition process, and labeled accordingly by auditory processing. Please note that in the normal sound group, we assumed that the normal cough (non-infectious cough) is caused by some environmental irritants such as dust and, ammonia that are usually found in an intensive farm, or it may due to other infectious pathogens, which are not PMWS, PRRS, or MH. Figure 3 shows a picture of a pig house complete with a stationary CCTV with an audio sensor. Figure 4 shows a typical example of automatic pig sound acquisition process using the continuous recording of a microphone marked with the red rectangle, which characterizes a non-infectious cough. Figure 5 shows the sound waveforms and spectrograms of the different cough sounds acquired from normal, PMWS, PRRS, and MH samples, respectively.

### 3.2. Pig Wasting Disease Cough Sound Detection Result

Firstly, we performed an identification test of the proposed mechanism between cough sounds due to pig wasting diseases and normal sounds. In our experiment, 300 pig wasting disease cough sound data (150 for PMWS, 120 for PRRS, and 30 for MH) and 200 normal sound data (50 for non-infectious coughs, 50 for screams, and 100 for grunts) were used. For the MFCC features, we used 24 frames per a pig sound and 12 cepstral coefficients with triangular filters (*i.e.*, part of the MFCC feature extraction process) and yielded 288-dimensional features (12 × 24 = 288) by using Matlab. The trade-off constant *C* in the Gaussian Kernel function was set to *C* = 0.01. The value of the parameter σ in the Gaussian Kernel function was chosen as 2.9.

We used three important formulas [28] in the evaluation of the performance of the proposed system: the cough detection rate (CDR), false positive rate (FPR), and false negative rate (FNR). They are given as follows:

(10) $$\text{Cough Detection Rate}\;(\text{CDR})=\frac{\sum_{i=1}^{n}T_{i}}{\sum_{i=1}^{n}I_{i}}\times 100$$

(11) $$\text{False Positive Rate}\;(\text{FPR})=\frac{\sum_{i=1}^{n}P_{i}}{\sum_{i=1}^{n}N_{i}}\times 100$$

(12) $$\text{False Negative Rate}\;(\text{FNR})=\frac{\sum_{i=1}^{n}F_{i}}{\sum_{i=1}^{n}I_{i}}\times 100$$

In the above equations, *I* is an individual infectious cough sound data, while *N* is normal sound data. *T* is infectious cough sound data that are classified as such by the system. *P* indicates normal sound data that are misclassified as infectious cough sound data. *F* indicates infectious cough sound data that are misclassified as normal sound data.

Our experimental results show that the average detection accuracy of the proposed system approached 94.0%, with FPR and FNR on average reaching 5.4% and 6.0%, respectively, when 70% of the normal data, randomly chosen, were used for training with a cross validation. Comparing our results with those of previous methods, our proposed system outperformed the results of previous methods (see Table 1). It should be noted that they all used different sound databases in their experiments.

### 3.3. Pig Wasting Disease Cough Sound Classification Result

Secondly, by using the cough sound data, we classified pig wasting diseases into three types: PMWS, PRRS virus, and MH. To measure the classification accuracy of the proposed system, the classification accuracy, precision, and recall were used as the performance measurements [29]:

(13) $$\text{Classification Accuracy}=\frac{\sum_{i=1}^{n}T_{i}}{\sum_{i=1}^{n}I_{i}}\times 100$$

(14) $$\begin{array}{cc} \text{Precision}=\frac{TP}{TP+FP}\times 100, & \text{Recall} \end{array}=\frac{TP}{TP+FN}\times 100$$

For a given class, the number of correctly classified objects is denoted True Positives (TP). The number of falsely identified objects is denoted False Positives (FP). The number of objects from a class that are falsely labeled as belonging to another class is denoted False Negatives (FN). Precision is the ratio of True Positives to True Positives and False Positives. This determines the number of correctly identified objects. Recall is the ratio of True Positives to True Positives and False Negatives. This determines the number of misclassified objects in a class.

The proposed system was realized by using Matlab, and SparseLab [30] was used as a sparse representation solver. Our experimental results show that the average classification accuracy of the proposed system was 91.0%, with precision and recall on average reaching 90.8% and 92.0%, respectively, when 60% of the data, randomly chosen, were used for training with a cross validation. The classification result is shown in Table 2. Note that, in literature, attempts to classify cough sounds due to pig wasting diseases have not been found; thus, a performance comparison can-not be made. In fact, the only one that comes close in literature, Ferrari *et al.* gave differences between healthy coughs and coughs made by *Pasteurella multocida* or *Actinobacillus pleuropneumoniae* [31].

## 4. Conclusions

Early detection of health anomalies is an important issue in the management of group-housed livestock. In particular, failure to detect pig wasting diseases in a timely and accurate way can become a serious limiting factor in achieving efficient reproductive performance. In this study, we introduced an efficient data mining solution for the detection and recognition of pig wasting diseases using sound data in audio surveillance systems. Primarily, the pig sound acquisition process was performed, and MFCC was extracted in the data preprocessing phase. The SVDD, which is often called an anomaly or novelty detector, was naturally used in the detection of pig wasting diseases sound. Finally, the SRC classified porcine wasting diseases into PMWS, PRRS virus, and MH.

From the experiments, we found that automatic detection and recognition of pig wasting diseases using sound data can be an efficient and economical solution. A combination of MFCC and SVDD can automatically detect pig wasting diseases using cough sounds at an accuracy level of 94%, and the SRC classified pig wasting diseases into PMWS, PRRS virus and MH at an accuracy average of 91.0%. As the sound data acquired from even a cheap microphone can detect pig wasting diseases accurately and economically, our method can be used either as a standalone solution or to complement other known methods to obtain a more accurate solution. Moreover, this study might be a confirmation that analysis of pig's sounds is a creditable method to understand the animal's present health condition.

In principle, once an off-line processing is done, it is no longer needed at on-line process in the proposed real-time system. As stated before, in one-class classification problems (SVDD), only the training data for the normal class are generally given, and after the training phase is finished, we are required to decide whether each test vector belongs to the normal or abnormal class. Therefore, even when new pig wasting diseases are added, the cost of updating or scaling is not required in the detection process of our proposed system. As to the SRC module, it can be easily adapted for incremental updating or scaling, even with a new pig wasting disease. All we need is a new column in a matrix *A* instead of retraining the entire system.

In future, we will consider the multi-modality of video and audio data in a pig house surveillance system. In fact, further testing and refinement of our proposed system, as needed, in commercial production settings are also warranted. That is, a complete real-time system, capable of incorporating the automatic detection and recognition of a pig's vocalization, is a part of our ongoing research.

## Figures and Tables

**Table 1.** Performance comparison between other cough sound detection methods and the proposed method.

	**Van Hirtum *et al***. [7]	**Exadaktylos *et al***. [19]	**Guarino *et al***. [20]	**Proposed** **Method**
CDR (%)	92.0	82.2	85.5	94.0
FPR (%)	29.0	12.0	13.4	5.4
FNR (%)	8.0	17.8	14.5	6.0

**Table 2.** Performance measurement of pig wasting diseases classification.

	**Precision (%)**	**Recall (%)**
PMWS	94.8	96.4
PRRS	92.0	97.8
MH	85.7	82.0
Average	90.8	92.0
